# Assessing Resilience of a Coastal Wetland to Relative Sea-level Rise for a Native American Tribe in Louisiana – Comparing Biophysical Prediction and Traditional Ecological Knowledge

**DOI:** 10.1007/s12237-026-01679-5

**Published:** 2026-02-20

**Authors:** Kelly M. San Antonio, Wei Wu, Matthew B. Bethel

**Affiliations:** 1 School of Ocean Science & Engineering, The University of Southern Mississippi, 703 East Beach Dr., Ocean Springs, MS 39564 USA; 2 Louisiana Sea Grant College Program, Louisiana State University, 232 Sea Grant Building, Baton Rouge, LA 70803 USA; 3 Department of Integrated Environmental Science, Bethune-Cookman University, Daytona Beach, FL 32114 USA

**Keywords:** Traditional ecological knowledge (TEK), Biophysical mechanistic model, Coastal louisiana, Coastal wetlands, Indigenous peoples

## Abstract

Coastal wetland predictions typically depend on biophysical models that incorporate geomorphological, hydrological, and vegetation dynamics. However, such models are rooted in scientific knowledge (SK), and can often exclude specific local environmental, cultural, and generational contexts. These aspects are better captured in traditional ecological knowledge (TEK), passed down through generations via oral histories. As TEK is context-specific and place-based, its comparison with SK can provide a more comprehensive understanding, especially to better predict coastal wetland vulnerability to relative sea-level rise (RSLR). In this study, we applied a biophysical mechanistic model to predict the impact of RSLR on Louisiana’s Terrebonne Bay wetlands, which are important for the subsistence and commercial livelihoods of the Pointe-au-Chien Indian Tribe (PACIT), but have experienced some of the highest rates of RSLR in the United States. The mechanistic model generated wetland predictions through 2100, using field measurements (vegetation productivity, soil pore-water salinity, total suspended solids, accretion rates), historic and current National Wetlands Inventory (NWI) wetland maps, and elevation data. We derived RSLR thresholds (beyond which wetland is lost dramatically) in 2050 and 2100 and applied the 2050 threshold to predict wetland change, which we then used to analyze the spatial vulnerability of coastal wetlands. Results indicate most wetland loss (~ 93%) will occur by 2075 under a high acceleration scenario, and were compared with previously co-produced TEK assessments to guide restoration prioritization, creating a GIS tool with broader insight than the SK or TEK data alone and providing a potential model for Indigenous-led climate adaptation planning.

**Supplementary Information:**

The online version contains supplementary material available at 10.1007/s12237-026-01679-5.

## Introduction

Coastal wetlands play an important role in enhancing the livelihoods in coastal areas and are effective in mitigating storm surge and flood risks (Fairchild et al., 2021; Costanza et al., 2008). In addition, “more than 75% of the commercial and 90% of the recreational harvest of fish and shellfish in the U.S. depend on coastal wetlands for food or habitat during some part of their life cycle”, highlighting their economic importance (*Value of Coastal Fisheries and Wetlands *n.d.). Louisiana contains 40% of the coastal wetlands in the contiguous United States, yet accounts for 80% of wetland loss (Bourne, 2000), driven by relative sea-level rise (RSLR, known as site-specific sea-level rise, accounting for both change in ocean’s surface and vertical land movement such as subsidence), geological compaction, extended dredged canals, a decline in sediments, and navigation and flood protection levees (Turner, 1997). Therefore, conservation and restoration are urgent priorities. To support this, landscape models have been developed to predict how coastal wetlands respond to RSLR and identify vulnerability hotspots (Wu et al. 2017a, 2020; Morris et al. 2003; Kirwan and Murray 2007). These scientific knowledge (SK) models, often also called academic ecological knowledge and western ecological knowledge, incorporate key ecological and biophysical data but often omit social and cultural context (Hatfield et al., 2018; Albuquerque et al., 2021; Souther et al.,. 2023), and can be reductionist and limited by the spatial resolution, scope, and vertical accuracy of input data.

Climate and environmental change historically and currently impact various cultures and communities disproportionately. Indigenous peoples in coastal systems, such as the Pointe-au-Chien Indian Tribe (PACIT) on the Louisiana Gulf Coast, are particularly sensitive to RSLR and climate change as it directly influences their resource-based livelihoods and homes (Wildcat, 2013; Bethel et al., 2022). Over generations, Tribe members have adapted to environmental changes such as RSLR, storms, and erosion, sustaining themselves in this place through traditional ecological knowledge (TEK) (Bethel et al., 2022). TEK is knowledge acquired through long-term observation and lived experience (Berkes 1993), and is typically delivered through oral history to better understand the connection between humans and nature (Bethel et al., 2022; Usher, 2000; Nadasdy, 1999). TEK differs from SK by centering on social, cultural, and place-based context (Gadgil et al., 1993; Berkes, 1993).

This type of data is place-based and generational; because of that, TEK is linked to culture, livelihood, and resiliency, making it inherently diverse and nuanced in its content and how it can inform (Thompson et al., 2020; Jennings et al., 2024). Additional insights could be derived when TEK is compared or integrated with the more quantitative SK models, that focus on current modeling and future prediction (Bethel et al., 2022; Moller et al., 2004), while reducing the limited scope and reductionist drawbacks that SK models have with counterbalance from the TEK’s broader context. This comparison or integration can produce complementary and overlapping views of the causes and potential consequences for change (Hatfield et al., 2018) because TEK is not a replacement for SK but a counterpart to it (Berkes, 1993; Suzuki & Knudtson, 1992). This complementary approach is emphasized due to the differing nature of the two knowledge systems, as TEK leans towards more qualitative data and holistic approaches, with data generated by resource users, while SK is defined in quantitative terms by science matter experts or practitioners (Barnhardt and Oscar Kawagley, 2005; Souther et al., 2023). As such, TEK provides relational, qualitative insights, while SK provides quantitative, mechanistic predictions. When combined, these knowledge systems may be able to highlight overlooked factors and support co-developed adaptation strategies through opportunities for shared dialogue, despite political, ethical, and logistical concerns in their combination (Albuquerque et al., 2021). There are several challenges involved in the integration or comparison of SK and TEK due to differences in data types and desire to ensure values are maintained. Ultimately though, SK and TEK can enrich resilience planning by identifying vulnerabilities, supporting cultural priorities, strengthening community ties, and fostering more equitable science partnerships (Bethel et al., 2022; Thompson et al., 2020; Moller et al., 2004; Hatfield et al., 2018).

The objectives for this study are: (1) predict short- and long-term RSLR impacts on Spartina alterniflora-dominated coastal wetlands in the Terrebonne Bay, near the PACIT, by 2100 using a biophysical model (SK), and (2) create a GIS tool that compares SK vulnerability predictions and previously collected TEK spatial assessments (vulnerability and sustainability) to better identify wetland vulnerability and guide restoration priorities in the Terrebonne Bay. This study does not attempt to integrate TEK directly into the mechanistic model. Instead, the purpose is to compare TEK vulnerability/sustainability assessments with SK-derived spatiotemporal predictions to identify areas of alignment and divergence relevant for PACIT adaptation planning.

## Methods

In order to compare the SK and TEK assessments, we first calibrated a mechanistic, biophysical model that spatially predicted coastal wetland change impacted by RSLR (Wu et al. 2017a, 2020). We used model predictions for 2050 and 2100 to derive RSLR thresholds indicating the maximum RSLR rates coastal wetlands can tolerate before experiencing substantial declines in structure and function. This SK model simulates elevation change-driven habitat change, informed by its simulated sediment accretion and erosion. The key inputs include elevation, initial coastal wetland distribution, the relation between vegetation productivity and elevation (and/or soil porewater salinity), and total suspended sediments (inorganic portion) as a proxy for sediment availability in the estuary. We then compared the model predicted wetland loss with the PACIT’s TEK-based vulnerability and sustainability maps, which identify areas of community concern and cultural priority. In evaluating the SK sea-level model output with the local TEK assessments, we engaged with multiple knowledge systems (Lake et al., 2018) to create new opportunities of examining coastal land loss through diverse perspectives. The creative strategy recognizes the benefit of local knowledge in applied research and participatory mapping approaches and ultimately helps to break down the barrier between social and scientific knowledge (Bethel et al., 2022; Laituri, 2011).

### Study Area

Coastal Louisiana is a dynamic landscape home to many Indigenous tribes, such as the PACIT. This state-recognized tribe inhabits Louisiana’s Terrebonne and Lafourche Parishes along Bayou Pointe-au-Chien, approximately 75 miles southwest of New Orleans (Bethel et al., 2022; Rivard, 2015; Yeoman, 2017). The PACIT descends from the regional Chitimacha, Biloxi, and Acolapissa tribes and has roughly 800 members who center around a key subsistence and commercial livelihood base of fishing, shrimping, oyster farming, hunting, and crabbing. Tribe members speak Indian French and continue to live and work with the surrounding lands and waters despite issues arising from climate change, such as land loss resulting in village migration further north and increased salinity driving away species traditionally used for fishing and trapping (Bethel et al., 2022; Ferguson-Bohnee, 2015; Rivard, 2015).

The PACIT and other tribes in this area face the combined effects of subsidence, continuous erosion, and RSLR, resulting in one of the areas with the highest rates of RSLR in the world at ~ 9 mm/year, or about an average of 3 feet/100 years (Parris et al., 2012; Bethel et al., 2022; Karimpour et al., 2013). The PACIT’s ecosystem-dependent livelihood base is a reason for their adaptability and understanding of the environmental changes they have experienced. This way of life, however, contributes to community vulnerabilities they face living in an area frequently impacted by RSLR, tropical storms, hurricanes, and issues brought about by climate change such as salinity inundation and land loss.

Terrebonne Bay, located in the Terrebonne Basin between the Mississippi River’s bird foot delta and the Atchafalaya delta, was part of a deltaic plain of the Mississippi River several thousand years ago and served as a main distributary within the last 1000 years (Coleman, 1981; Karimpour et al., 2013; Penland et al., 1987; Wang et al., 1993). The bay is surrounded by wetlands comprising a large proportion of saltmarsh habitat, which is a highly productive system of primary production and carbon storage (Chmura et al., 2003; Hill & Roberts, 2017; Karimpour et al., 2013). The saltmarshes of Terrebonne Bay are dominated by *Spartina alterniflora*, with smaller patches of *Avicennia germinans*,* Spartina patens*,* Distichlis spicata*, and *Juncus roemarianus* also observable (Hill & Roberts, 2017).

Terrebonne Bay rarely sees clear water and receives little to no fluvial inflow or external sediment deposition. As such, the sediment and turbidity changes here are attributed to the processes that occur within the wetlands and the bay, such as bay bed erosion, wave activities, cold fronts, and storm influences (Karimpour et al., 2013). The coastal wetlands of this region experience low amplitude tides (~ 0.3 m; Hill & Roberts, 2017) that are diurnal, as well as irregular floodings, with inundation more influenced by wind and larger-scale meteorological activities than by tides (Childers and Day, 1988; Turner, 2001; Schutte et al., 2020; Wang et al., 1993). Using the Coastwide Reference Monitoring System (CRMS), the average 2022 and 2023 salinity measured around this study’s field sites was reported as 21.04 ppt and 21.00 ppt respectively, with an average water temperature of 22.96 °C (2022) and 23.84 °C (2023) (Coastal Protection and Restoration Authority (CPRA, n.d.).

### Field Work

**Fig. 1:**
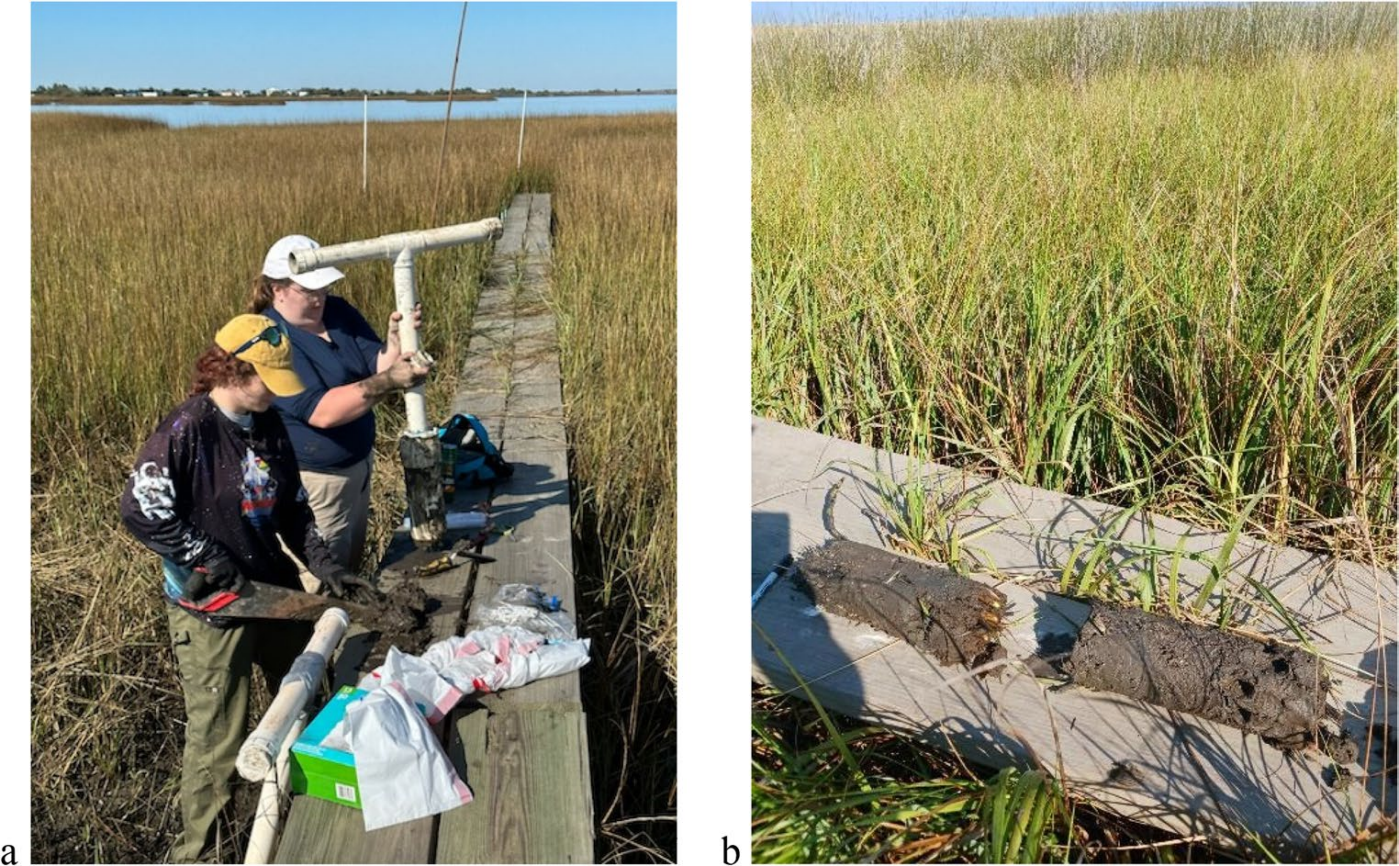
Collection of the biophysical data used in the mechanistic RSLR model **a** Belowground biomass extraction with a PVC soil core tool and a handsaw to section samples into 5 cm increments. **b** Soil cores ready for sectioning

**Fig. 2:**
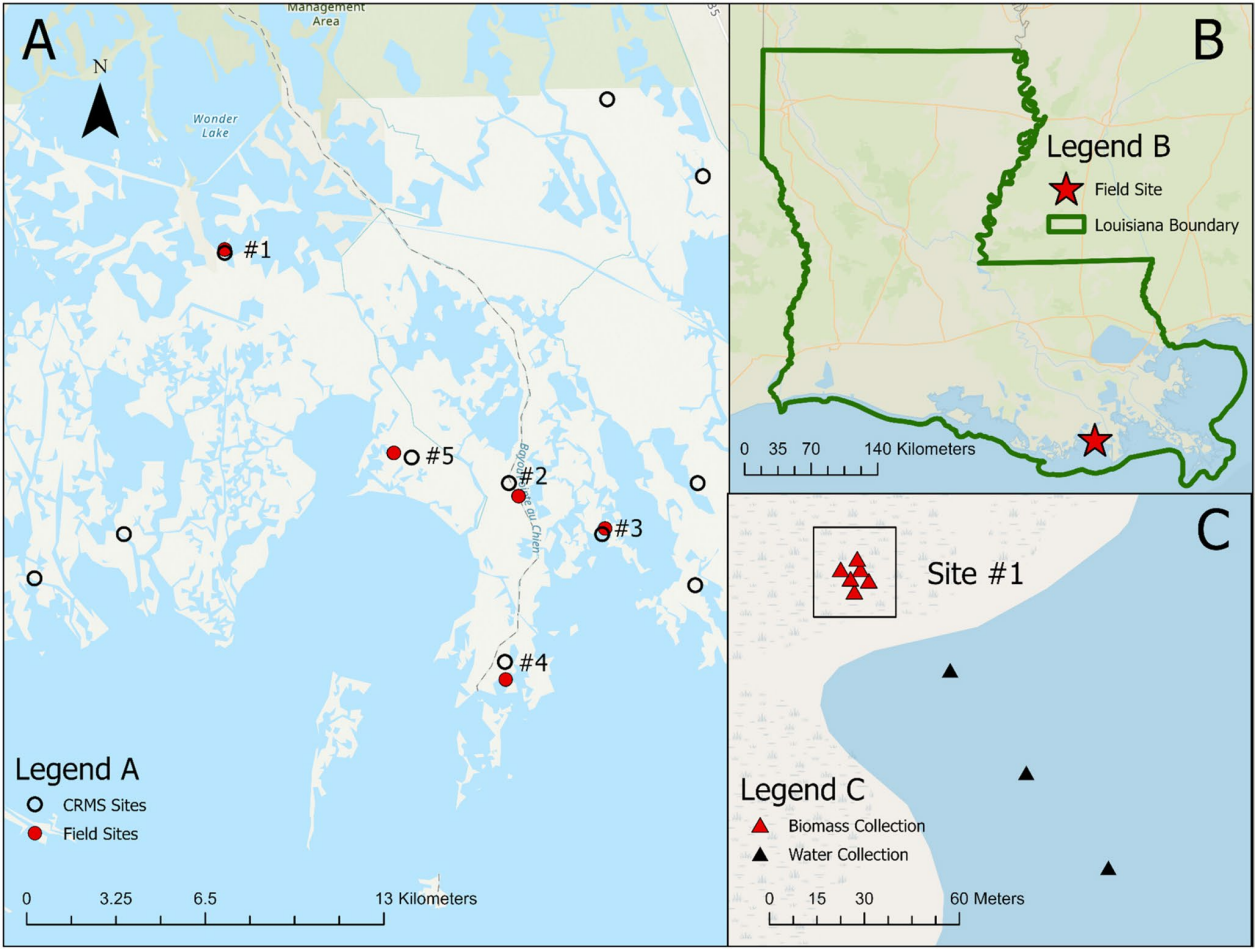
**a** Map of southern Louisiana’s Terrebonne Bay with five sample sites (red circles) in proximity to the CRMS sites (open circles). **b** Study location within Louisiana. **c** Sample site location #1 illustrating TSS water collection (black triangles) and the biomass collection (red triangles)

 To collect the biophysical data needed for the mechanistic RSLR impact model (vegetation productivity indicated by peak-season live biomass, soil pore-water salinity, and total suspended solids (TSS)), field work was conducted in the coastal wetlands of the Terrebonne Bay close to the PACIT’s territory. Field work commenced at the end of the growing season in December 2022 and again in September 2023, during peak saltmarsh growing season (Figs. 1 and 2b). Five sites were chosen near CRMS stations to pair with long-term monitoring data (Fig. 2a), with input from the PACIT partners on specific site selection and navigation to the sites. Above- and belowground biomass was collected at five sites. At each site, two parallel 3-m transects were set up perpendicular to the coastline to capture small-scale elevation variation. At each transect, the three subsites about 1 m apart were established and duplicate samples were collected at each subsite (Fig. 2c).

Spatial coordinates were measured at each subsite along the transects. Aboveground biomass was collected using 25 × 25 cm quadrats at each subsite along the transects, cutting the entirety of live and dead surface biomass within each quadrat, placed in bags, and stored in coolers to stave off decomposition. After removing the aboveground biomass, belowground biomass was then dug up at the same location using 30 cm long 4-inch (10.16 cm) diameter soil cores, which were then separated into 5 cm increments using a handsaw and then stored in a cooler (Fig. 1b). Bags were placed in a cooler until returned to the lab and then stored in 4 °C refrigerators for later processing. Additionally, when approaching each site from a boat, three water samples were collected using one-liter bottle grabs, with coordinates recorded. The water samples were filtered in the lab later using glass microfiber grade GF/F filter paper that retains particles down to 0.7 μm to derive the total suspended solids (TSS) (Fig. 2c).

### Laboratory Processing

The processing of above- and belowground biomass started by separating live from dead biomass. For aboveground biomass, processed within one week of collection, green leaves were designated as live while brown and yellow leaves were designated as dead. Likewise, for belowground biomass, roots that were white, turgid, and floating in water were “live” while beige and brown roots that lacked turgidity and ability to float were “dead” (Wu et al., 2020; San Antonio et al., 2025). Once biomass was separated and placed into oven-safe aluminum trays, wet weights were taken along with tray weights. Trays were then dried in an oven at 75 °C for three to four days until constant weight was reached. Dry weights were taken and used to calculate live above- and belowground biomass in units of g/m^2^.

To calculate porewater salinity, 10 g of wet soil from each depth and replicate of each subsite were placed in oven-safe trays and dried in an oven at 75 °C until constant weight, similar to the biomass process. After the soil dried, the difference between the wet and dry weight was calculated as the soil moisture content. Subsequently, 20 milliliters of distilled water was added to each sample of dry soil and samples were then mixed thoroughly to allow salts adsorbed onto the soil to be dissolved in water. After mixing, the solids settled for at least an hour, after which a refractometer was used to measure soil salinity in units of parts per thousand (ppt). Using this data, porewater salinity was calculated with a mass balance equation (Eq. 1):

1 $$S_{porewater}\left(ppt\right)=S_{20ml}\left(ppt\right)\times\frac{20\left(ml\right)}{\left(W_{wet}-W_{dry}\right)\left(g\right)/\left({\displaystyle\frac{1g}{ml}}\right)}$$

Where $\:{S}_{porewater}$ denotes salinity in soil porewater, $\:{S}_{20ml}$ denotes measured salinity, W denotes weight, $\:\frac{1g}{ml}$ is density of water.

To calculate TSS from the water samples, pre-weighed glass fiber filters were placed in a furnace at 400 °C for four hours to remove organic content. Once ashed, the filters were weighed again with an analytical balance. A vacuum pump was used to filter water samples, with duplicates from each sample bottle passed through the prepared filter papers, while recording the volume filtered for each. The wet filters were then dried in an oven at 75 °C overnight to completely dry out and remove the remaining water. Once dry, the filters with the solids were weighed to collect filter paper and total solids weights. The dried filter papers were then placed back in the furnace for four hours and weighed again to derive the inorganic sediment weights only. We then calculated inorganic TSS, in units of mg/L, using the following equation (Eq. 2):

2 $$\begin{array}{l}{TSS}_{inorganic}\left(\frac{mg}{L}\right)\\=\frac{{W}_{after\:filtering\:\&\:ashing}\:\left(mg\right)-{W}_{filter\:pape\:after\:ashing}\left(mg\right)}{{V}_{water\:filtered}\:\left(L\right)}\end{array}$$

### Model

#### Model and Data Description

A landscape model (Wu et al. 2017a, 2020) was applied to predict coastal wetland change based on average of TSS data in 2022 and 2023 and peak biomass data in 2023. This model accounts for vegetation processes and hydrodynamics to make predictions of coastal wetland distributions under future scenarios of RSLR with annual time steps. It is a two-dimensional and mechanistic model that uses elevation as the key driver for vegetation productivity and landscape change while also simulating accretion and erosion (Eq. 3). The model for this study uses biophysical inputs to predict elevational change and the conversion of coastal wetlands to estuarine open water once the elevation is below the mean low water level on 2 × 2 m cells, the spatial resolution of LiDAR derived elevation used in this model (Wu et al., 2020).

3 $$\:{Elev}_{t\:}={Elev}_{t-1}-\:{RSLR}_{t\:}+\:{Acc}_{t}\:-\:{Ero}_{t}$$

Where *Elev* denotes elevation (m), *RSLR* denotes relative sea-level rise rate (m/yr), *Acc* denotes accretion rate (m/yr), and *Ero* denotes erosion rate (m/yr). The subscript *t* denotes time (adapted from Wu et al. 2017a).

The model inputs include 2011 LiDAR-derived elevation data from the U.S. Army Corps of Engineers, with a spatial resolution of 2 m, vertical datum of NAVD88 and a vertical accuracy of 8 cm. The current RSLR rate is 9.1 mm/year (Herbert et al., 2021). Sediment bulk density was acquired through the Coastwide Reference Monitoring System (CRMS), averaged from the field data as 0.289 g/cm^3^ for the Terrebonne Bay, Louisiana. Coastal wetland maps from 1989 to 2023 were available from the National Wetland Inventory datasets upon request. Both organic and inorganic TSS, and above- and belowground biomass were collected in-situ, with mineral sediments in the water column and belowground biomass as the primary contributors to inorganic and organic accretion, respectively (Wu et al. 2017a, 2020). For more details on the model and a diagram of the mechanisms, see Wu et al. 2017a.

The output of this spatial SK model produces maps of predicted coastal wetland distribution across time, identifying areas of vulnerability defined as areas susceptible to loss or subsidence. Based on the timelines of predicted wetland loss, we compared biophysical model predictions with previously collected TEK spatial data (Bethel et al., 2022). The TEK dataset included in this comparison was co-produced with PACIT members through participatory mapping, field-based interviews, and focus group dialogues (Bethel et al., 2022). PACIT representatives granted permission for its use in this follow-on modeling study. TEK is not treated as an extractive dataset but as knowledge embedded in cultural relationships and responsibilities; therefore, all uses were reviewed collaboratively with Tribal partners to ensure cultural appropriateness. The comparison goal was to understand more comprehensively the vulnerability and sustainability of coastal wetlands in response to RSLR which is key to inform and identify areas that need urgent protection from both SK and the PACIT’s perspective.

The original TEK assessment output, developed through complete partnership with the PACIT throughout the entire assessment process, the co-produced using sustainability and priority factors, including spoil banks and levees which experience the least erosion, and vulnerability factors, such as elevation-based inundation under RSLR, canals dredged for oil/gas exploration, historical land loss, and fragmentation. These factors were identified by PACIT members through a series of field-based interviews, participant observations, and focus group meetings, and transcribed into parent and child codes to be used in the mapping process, with emphasis given to select factors based on TEK-experts’ guidance (Bethel et al., 2022). Sustainability factors are defined as factors that increase the wetland’s sustainability or adaptability to climate change and RSLR. Vulnerability factors are defined as factors that increase or accelerate the wetland loss. An area or topographic feature with a high sustainability rating (3) means it contributes greatly to the stability of coastal marshes and the PACIT community while a high vulnerability rating (3) denotes an area or feature that contributes greatly to the community’s vulnerability to coastal hazards and highly vulnerable to rising waters and subsequent subsidence (Bethel et al., 2022). This information is largely based on observed past trends and current conditions. For more information on how the TEK assessments were derived and subsequent mapping, see Bethel et al., 2022.

In order to compare the the SK and TEK predictions, we focused on vulnerability from the TEK perspective and compared it with the vulnerability derived from the biophysical model. TEK sustainability factors were added separately for additional comparison. By comparing RSLR SK wetland predictions with existing TEK predictions, alignment and discrepancies between the two can be evaluated and lead to more informed projections of coastal wetland loss, lending additional confidence to region-specific adaptation strategies and decision-making for the Tribe’s members and land managers.

#### Input Data and Functions

Due to nested structured sampling design, linear mixed effects models were applied to simulate above- and belowground biomass as a function of elevation, a proxy for inundation, and soil porewater salinity with sample site as a random factor using the “lmerTest” package in R (Kuznetsova et al., 2017). Models were compared with different combinations of the covariates based on Akaike Information Criterion (AIC), and the model with the lowest AIC was selected as the best predictive model (Wu et al. 2017b, 2020). The best model had only elevation as the covariate, likely as soil porewater salinity measurements, for example, are just static snapshots of conditions at time of collection and did not contribute strongly to model predictions. A mixed effects model was developed to derive the spatial distribution of inorganic total suspended solids, relating the inorganic TSS with spatial Northing in UTM projection with the site as the random factor. Like the other mixed effects models, this model was selected because it had the lowest AIC value compared to the model with both northing and easting as the fixed factors.

#### Model Calibration

The accretion rate was derived by combining the contribution from water column-borne inorganic matter settled or intercepted by aboveground biomass and organic contribution from belowground biomass. It is calibrated using the measured accretion rates at the CRMS sites nearby. The model was calibrated further by comparing simulated 2023 water velocity to the measured data in the literature. One study found that velocity rates for the Terrebonne Bay ranged from − 0.5 m/s to 0.5 m/s (Wang et al., 1993), with the model’s simulated velocity ranging from 0 m/s to 0.094 m/s. The model was considered calibrated when simulated values were in range of the associated target ranges.

Additionally, the accuracy of the simulated wetland distributions in 2023 was evaluated in comparison to the 2023 NWI data (considered as ground truth). A modified kappa, which accounts for persistent land cover, was applied to evaluate land cover change more accurately (van Vliet et al., 2011; McHugh 2012). A large value of a traditional kappa statistic generally results from the majority of land remaining unchanged as it does not separate accuracy due to persistence and change, while the modified kappa specifically evaluates the accuracy of modeled changes. Furthermore, the figure of merit was calculated as the ratio of hits to the total of hits, misses, and false alarms, serving as an additional metric to assess the model’s accuracy in simulating land changes (Pontius et al., 2011; Wu et al., 2015, 2020). Hits represent reference land changes correctly simulated as changes, misses indicate reference changes incorrectly simulated as persistence, and false alarms refer to reference persistence incorrectly simulated as change.

#### Model Scenarios and Comparing SK with TEK

With the calibrated RSLR model, the model was run under various RSLR rate scenarios, from RSLR rates of 9 mm/year to 20 mm/year to identify the threshold of RSLR. These value ranges were selected using the current rate as the starting point with a wide range to allow for various predictions, given that RSLR predictions for this region are also varied (Marshall, 2013). The RSLR threshold was defined as the rate of RSLR that results in a wetland area being lost midway between the area under the current RSLR rate (9.0 mm/yr) and the area under an RSLR rate of 20 mm/yr for a specific year (2050, 2075, and 2100). Note that the RSLR threshold becomes smaller for years farther into the future, because it takes a higher rate of RSLR to cause wetland collapse in the near term than in the more distant future.

To evaluate the effect of accelerating RSLR instead of constant RSLR, we further ran the model under scenarios with one low acceleration rate and one high acceleration rate. In the low acceleration scenario, the RSLR rate starts at 9 mm/year in 2023, increases to the 2100 RSLR threshold value by 2050, and then remains constant. In the high acceleration scenario, the RSLR rate also starts at 9 mm/year in 2023 but increases to the 2050 RSLR threshold (larger than 2100 RSLR threshold) by 2050, after which it remains constant. Using the predicted maps from just the high accelerating scenario, we identified wetlands projected to be lost by 2050, 2075, and 2100. We then assessed which TEK-based vulnerability and sustainability classes these lost wetlands fall into. We expected that the TEK-based medium and high vulnerability areas will align with wetlands lost by 2050. The wetland areas that would be lost by 2050 were classified as the most vulnerable areas (3), wetland areas that would be lost by 2075 and 2100, as medium vulnerable areas (2), and wetlands that would be persistent by 2100 as least vulnerable areas (1).

Additionally, to assess how restoration methods (e.g., living shorelines, freshwater diversions) might mitigate wetland loss, we modified the model’s accretion and erosion parameters and made predictions of coastal wetland change. These adjustments represented enhanced accretion and/or reduced erosion under different restoration scenarios.

## Results

### Biophysical Data

**Fig. 3:**
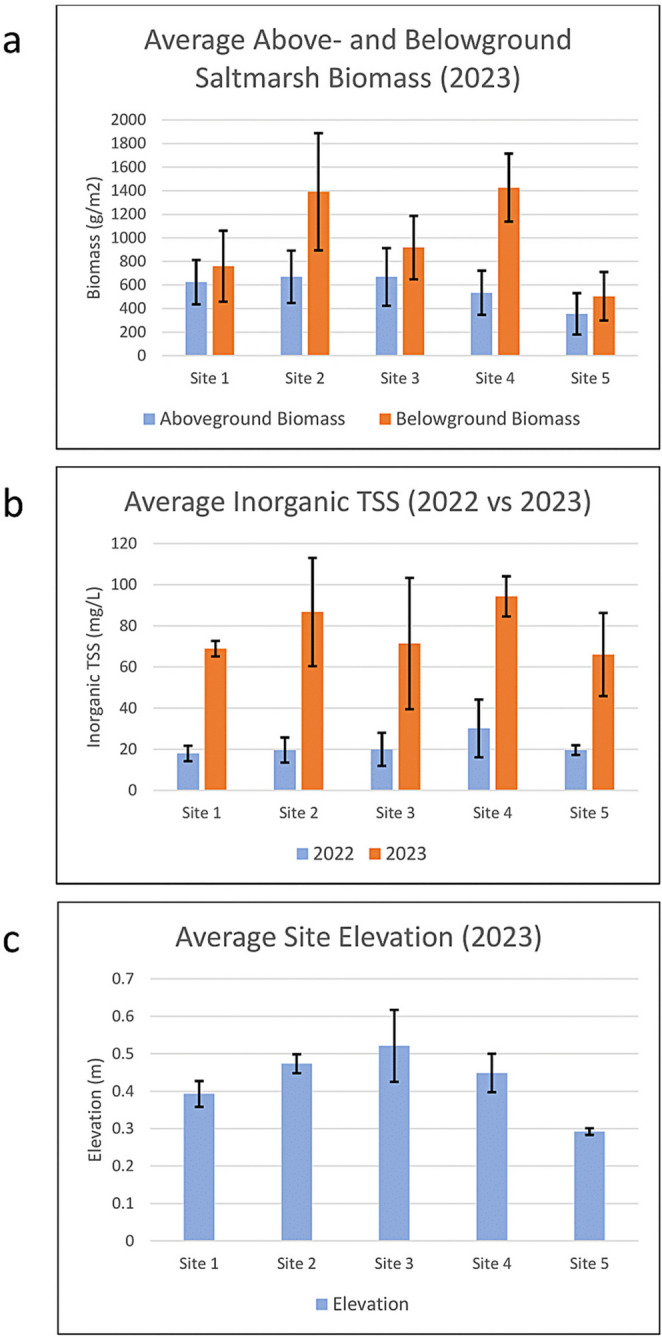
Average biophysical data by site with standard deviation error bars. **a** Above- and belowground biomass (2023). **b** Inorganic TSS (2022 vs. 2023). **c** Elevation for the sites of 2023 derived from the 2011 LiDAR

Aboveground biomass between sites was fairly uniform, showing slightly smaller values in sites 4 and 5 (Fig. 2a), which may indicate that aboveground biomass more exposed to the Terrebonne Bay is slightly smaller than aboveground biomass located further inland or more protected in general (Fig. 3a.). Belowground biomass was consistently higher than aboveground biomass, exceeding it by more than twofold at sites 2 and 4 (Fig. 3a).

TSS in 2022 is much lower than TSS collected in 2023, likely related to preceding precipitation events in 2022 and seasonality (Fig. 3b). Elevation is fairly consistent across sites, ranging from 0.3 to 0.5 m (Fig. 3c). Site 3 displayed the highest elevation as well as the largest standard deviation between samples subsites at that site, potentially indicating that this site exhibited the largest change in slope elevation along the transects. See Figure A1 in the appendix for spatial pattern of elevation across the study area.

### Calibration

The calibrated model, producing simulated biophysical values (accretion, erosion, etc.) within range to the CRMS and literature values, produced a kappa statistic of 0.40 for the model simulated 2023 wetland map. This indicates a fair agreement between the model predictions and observed data. This kappa statistic is the product of Ktransition and Ktranslocation. Ktransition represents matches of quantities of transitions between reference and simulated maps and ktranslocation assesses matches of locations (van Vliet et al., 2011). Additionally, the figure of merit, 0.32, is similar to the performance of the mechanistic model previously calibrated at the Pascagoula River delta (Wu et al., 2020). This assessment gives confidence that the model can adequately predict coastal wetland change under RSLR.

### Threshold Identification

**Fig. 4:**
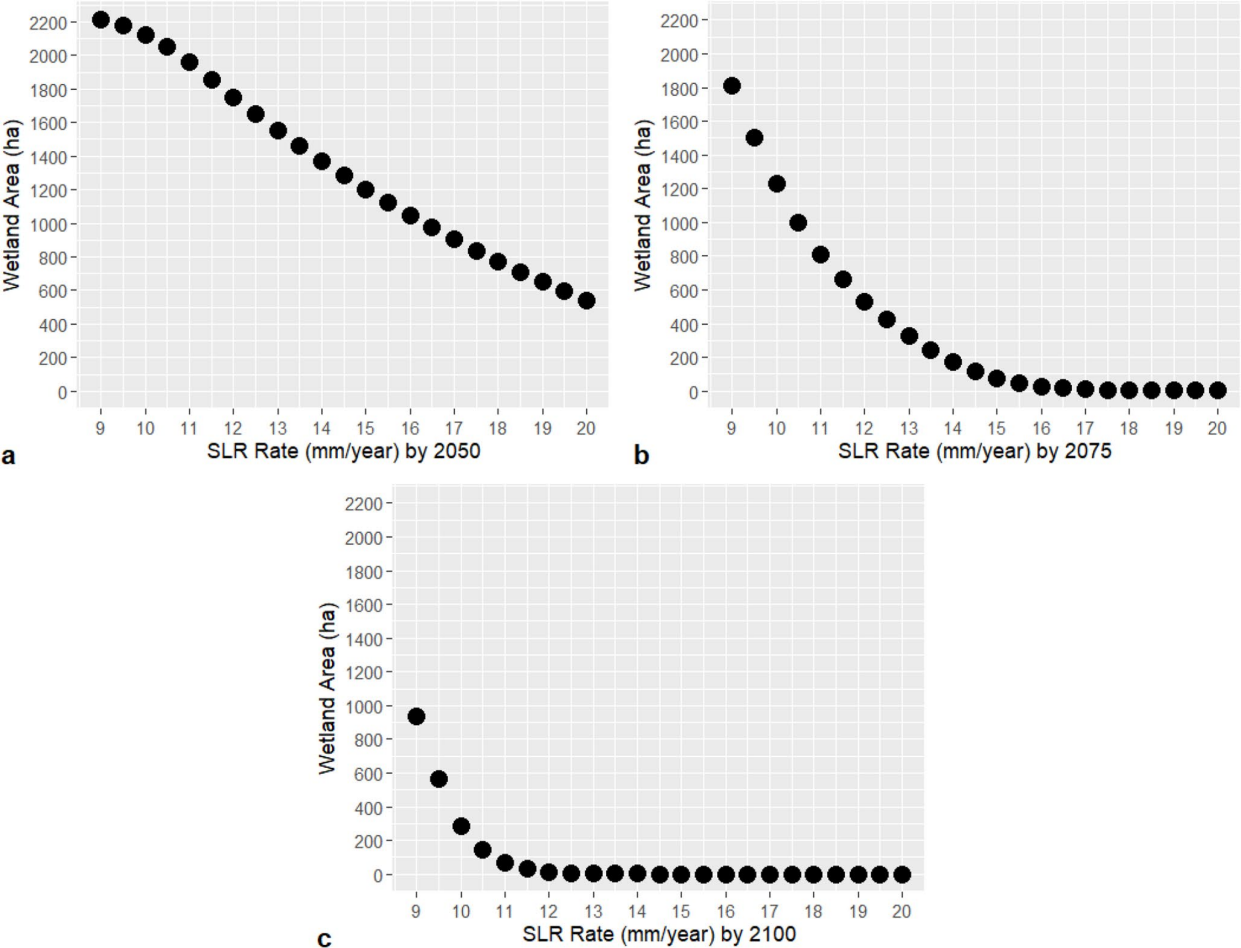
Predicted wetland area (hectare) for years 2050, 2075, and 2100 vs. RSLR rate

The model threshold was determined to be 14 mm/yr for 2050, 10.8 mm/yr for 2075, and 9.7 mm/yr for 2100 (Fig. 4). Based on the thresholds, the model was run using two accelerating RSLR rates. The high RSLR acceleration scenario begins at the current rate of 9 mm/yr in 2023, increasing linearly to 14 mm/yr by 2050, which represents the RSLR threshold for that year, and then remains constant at 14 mm/yr thereafter. The low RSLR acceleration scenario also starts at 9 mm/yr in 2023 but increases linearly to 9.7 mm/yr by 2050, corresponding to the RSLR threshold for 2100, and remains constant at 9.7 mm/yr thereafter.

### Wetland Predictions Under Accelerating RSLR

**Fig. 5:**
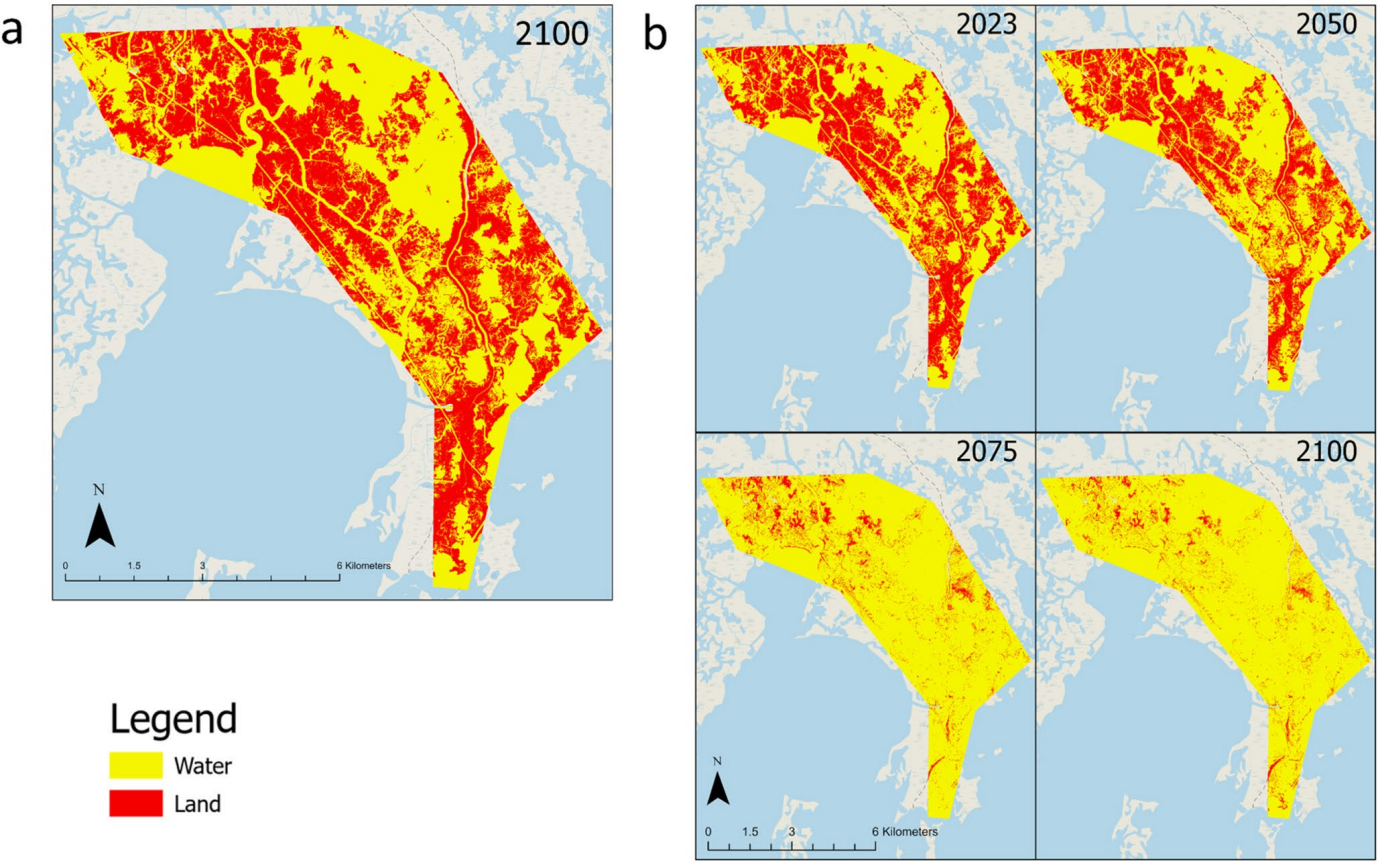
**a** Projected land and water by 2100 under the low RSLR acceleration scenario, **b** Projected land and water for 2050, 2075, and 2100, under high RSLR acceleration scenario, starting at 2023

**Fig. 6:**
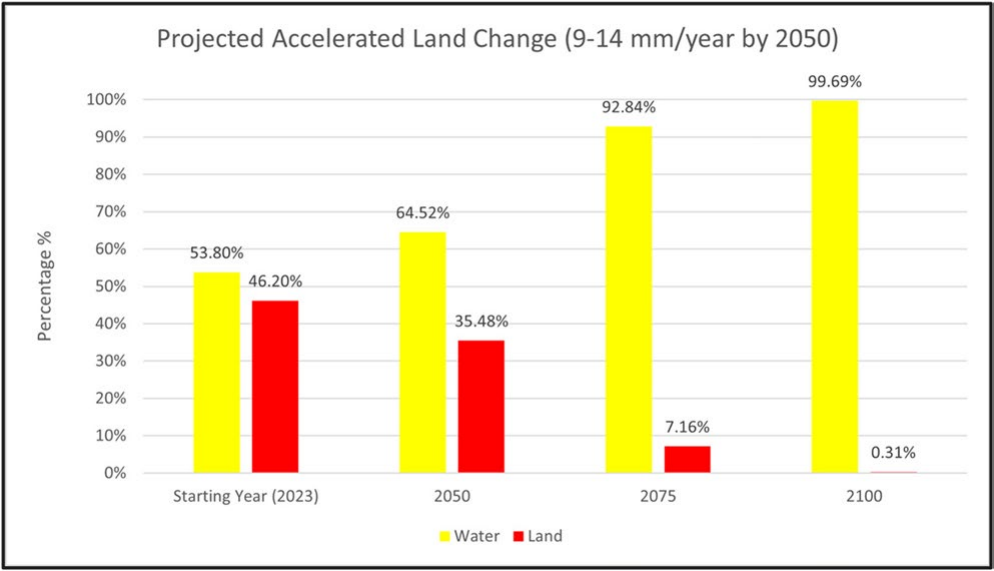
Percentage of total areas (5,336 ha) under accelerated RSLR. The starting year for the study area displayed 2,465 hectares of land and 2871 hectares of water

In the low-accelerating RSLR rate scenario, very little land is predicted to be lost by 2100, with remaining land predicted to be 2386 hectares, compared to the wetland area from 2023 of 2465 hectares (Figs. 5a and 6). Since the implemented RSLR rate only reaches the 2100 threshold, it is expected that most wetland loss will not occur until after 2100 (Fig. 4). Under the high-accelerating RSLR rate scenario, wetland loss is largely concentrated in the middle of the study area from 2023 to 2050, with land mass dropping from 46.20% to 35.48% (Figs. 5b and 6). However, by 2075, the wetland predictions show more drastic wetland loss, with the largest of the remaining land located in the north of the study area (Fig. 5b). This larger amount of loss between 2050 and 2075 scenarios drops the land’s area from 35.48% to 7.16% by 2075 (Fig. 6). Between 2075 and 2100, there is less loss, as most of the remaining land is already gone (Figs. 5b and 6), indicating that most of the wetland loss is predicted to occur between 2050 and 2075, consistent with the more aggressive RSLR scenario that reaches the 2050 threshold by mid-century. Given that there is very little predicted wetland loss with the low-accelerating scenario, our comparison with the TEK assessments focused on the high-acceleration RSLR predictions.

### Comparison of Coastal Wetland Vulnerability Based on TEK and the Biophysical Model

**Fig. 7:**
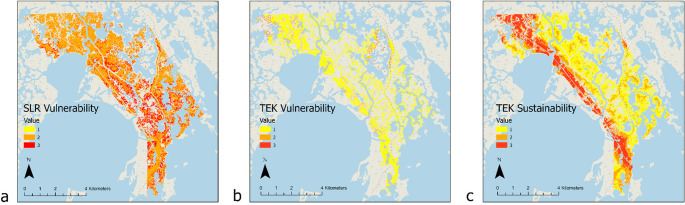
RSLR vulnerability assessment **a** based on the biophysical model predictions, the TEK vulnerability assessment **b**, and the TEK sustainability assessment **c**. The classes 1 (yellow), 2 (orange), and 3 (red), represent low to high vulnerability/sustainability of coastal wetlands. Note the smaller area of data availability for the TEK vulnerability than for the RSLR biophysical model predictions, as some data was not included in the TEK assessment

**Fig. 8:**
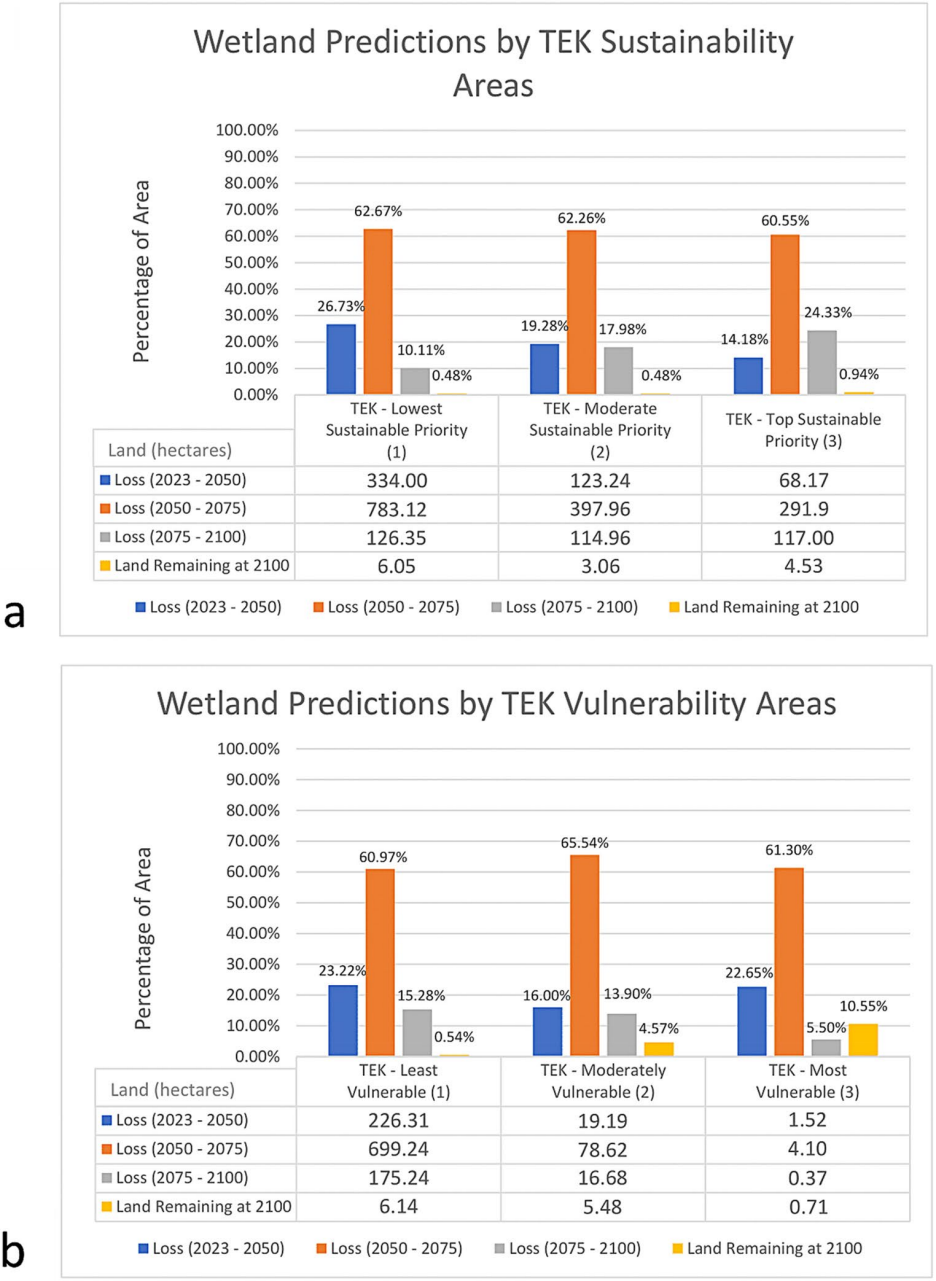
**a** Areas (hectares) of wetland loss (SK model) in TEK vulnerability classes, **b** and in TEK sustainability classes

Figure 7a-c. shows how the assessment methods (the SK model and the TEK assessments) vary in the distributions of sustainability areas. The majority of the wetlands of the RSLR vulnerability map are classified as “moderately” vulnerable, while the majority of the wetlands in the TEK vulnerability assessment are designated as “low” vulnerability (Fig. 7a-b.). This shows that the RSLR SK vulnerability model places the largest emphasis on class 2, suggesting that the most vulnerable wetlands will experience potential loss not in the immediate future (i.e. 2023–2050), but after mid-century. The predicted SK vulnerability differs from the TEK vulnerability assessment, which shows that most of the wetland area is the lowest vulnerability priority (class 1). Despite the discrepancies, we found that the largest proportion of coastal wetlands projected to remain after 2100 falls within the highest sustainability class (Fig. 8a). The discrepancy and consistency in the vulnerability assessment highlights the combined benefits of the RSLR SK modeling approach and TEK assessment as both a validation and complementary tool.

The apparent vulnerability discrepancy arises primarily from the high-accelerating RSLR scenario, which is likely to represent the future that has not yet been experienced by the PACIT community. As a result, SK and TEK offer different perspectives on vulnerability because they interpret RSLR scenarios in different ways. In contrast, under the low-accelerating RSLR scenario, SK predictions align closely with TEK assessments, where the majority of coastal wetlands persist through 2100 (Fig. 5a) and would fall into the low-vulnerability category.

The TEK sustainability assessment has more evenly distributed sustainability areas across different classes, which detail priority areas for sustainability and potential priorization, than the two vulnerability assessments, indicating that most of the top sustainability areas lie along a major bayou (Bayou Pointe au Chien) that bisects the northwest to the southeast of this study area (Fig. 7). When the top sustainability areas are isolated (class 3), the specific differences between the SK and TEK vulnerability assessments (future predictions vs. static historic snapshot respectively) within this class highlights their potential as complementary findings and approaches to sustaining the Tribe’s priority areas within the high sustainability zones (Table 1).

**Table 1:** Class percentages of the two vulnerability assessments (RSLR and TEK), within the tribe’s top sustainability areas (TEK sustainability class 3)

Vulnerability Class Percentages within TEK Sustainability’s Priority Class 3			
Vulnerability Type	Class 1 (low)	Class 2 (medium)	Class 3 (high)
RSLR Vulnerability	1.06%	83.74%	15.20%
TEK Vulnerability	92.01%	7.60%	0.39%

Since the total area of low, medium, and high TEK vulnerability classes varies, percentages were used for comparison in Fig. 8a. This figure displays an alternate way to compare assessment methods, showing predicted RSLR wetland loss and remaining land by 2100 compared within the TEK vulnerability and sustainability areas. Modeled wetland loss and TEK sustainability class has some similarities, with the predicted wetland loss decreasing with higher sustainability class and the least predicted loss in the highest sustainable priority area (class 3) (Fig. 8a). This supports the alignment between TEK and biophysical model predictions. TEK assessments of sustainability take into account spoil banks, which experience the least erosion. Additionally, this indicates that there may be sufficient time to prioritize restoration of these sustainable wetland areas before predicted major wetland loss begins after 2050. In the high TEK vulnerability class, 89% of coastal wetlands are predicted to lose to RSLR by 2100, while losses increase to 95% and 99% in the medium and low vulnerability classes, respectively. Although the biophysical models did not explicitly include spoil banks, the positive relationship between biomass (above- and belowground) and elevation suggests reduced vulnerability at spoil bank sites with higher elevation.

The discrepancy is again apparent in vulnerability comparison. Predicted loss is more unevenly dispersed across TEK classes, with the greatest loss evident in the TEK vulnerability class 1 and the least loss visible in class 3, as most of the TEK vulnerability data lies within class 1 (Fig. 8b). Overall, the percentage distribution of vulnerability levels from biophysical predictions appears consistent within each TEK vulnerability class. However, SK predicts a higher percentage of coastal wetland loss by 2075 in the higher TEK vulnerability class (3, 84%) compared to the lower TEK vulnerability class (2, 82%), demonstrating some degree of consistency between TEK-based and biophysical vulnerability assessments. It is important to note, however, that most of the predicted wetland loss by 2075 reflected by the percentages is due to the use of the 2050 RSLR threshold scenario, i.e. high accelerating scenario with major wetland loss after 2050, in the biophysical model predictions.

### Potential Restoration and Mitigation Effects Via RSLR Modeling

**Fig. 9:**
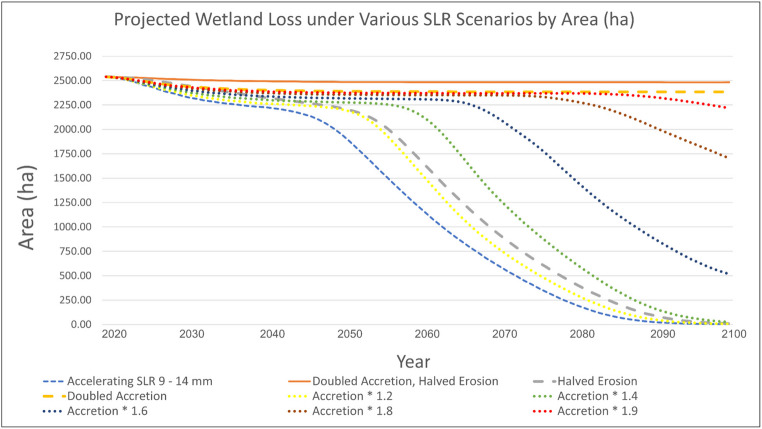
Predicted wetland area (hectares) to 2100 under various accretion and erosion scenarios with high RSLR acceleration

In an aggressive restoration scenario where the effort doubles accretion and halves erosion, there is almost no wetland degradation (Fig. 9: orange solid line). The three restoration effort scenarios underneath that line (doubled accretion, accretion *1.9, and accretion *1.8 respectively, e.g. freshwater diversions) show minimal but increasing wetland loss. However, in restoration scenarios that only increase accretion by *1.6, wetland loss begins to occur more drastically, indicating that only 27% of wetlands would remain by 2100 (Fig. 9). All of the other scenarios (accretion*1.4, halved erosion, and accretion*1.2) result in similar wetland loss to the model without restoration effort, highlighting that restoration that cannot increase accretion rate by more than half, and efforts that are solely focused on reducing erosion (living shorelines) may not have much impact on reducing wetland degradation at the large scale (Fig. 9). These results clearly indicate that restoration success hinges on enhancing accretion rates and should be a key consideration during decision-making and implementation.

## Discussion

This study demonstrates how combining SK and TEK provides complementary perspectives on wetland vulnerability and resilience for the PACIT. As the low-accelerating RSLR model is what the tribe has historically witnessed and is currently experiencing, the predicted minimal wetland loss by 2100 indicates high alignment with the TEK vulnerability, as those areas would likely fall into class 1, least vulnerable (Figs. 5a and 7). This corroboration reflects the community’s lived experience, which is more consistent with the low-accelerating scenario. On the other hand, the high-accelerating RSLR scenario, while not a scenario that has been experienced yet in this area, shows more discrepancy and can provide better guidance on areas that will experience such high-acclerating RSLR (Fig. 6). While TEK is grounded in past and present observations, SK predictions enable exploration of multiple RSLR scenarios, particularly the high-acceleration scenario that the community has not yet experienced but may face in the future, making these projections valuable for community planning. The high-acceleration RSLR scenario aligns more closely with TEK priorities, highlighting widespread mid-century loss (2050–2075) as the critical adaptation window. These areas of overlap suggest that TEK-based priorities within sustainability areas often coincide with locations that the SK model identifies as relatively less vulnerable under high-acceleration scenarios.

Several factors influencing data collection and model input could be altered to improve landscape predictions. Updated input data, specifically newer LiDAR data, would improve model predictions and reduce uncertainty. Additionally, the sample sites are distributed more towards the southern half of the study area, due to our attempt to align our sampling sites to the CRMS station locations to take advantage of CRMS’ long-term data (accretion rate). Increasing the number of sites within the Terrebonne Bay study area would help to understand modeling accretion/subsidence dynamics better. Organic and inorganic accretion is key for coastal wetlands to keep up with RSLR; thus, if the accretion rate is lower than RSLR, the coastal wetlands will be lost to water over time (Stevenson et al., 1986). This region experienced a drier-than-normal years in 2022 (~ 0.14 in precipitation/month) and 2023 (~ 0.13 in precipitation/month) when compared to 2021 (~ 0.17 in precipitation/month) and 2024 (~ 0.17 in precipitation/month) (National Weather Service, 2026), and as such, the biomass we collected may have caused our RSLR model’s predictions to overestimate wetland loss. If the more aggressive RSLR scenario comes to pass, the major predicted loss of wetland by 2075 is a larger cause for concern, indicating that most of the predicted wetland loss will not occur close to the end of the century, but between 2050 and 2075. The prediction is not surprising, as the RSLR model started with the current rate of 9 mm/yr and increased to 14 mm/yr by 2050, which was the consistent rate until 2100. The collapse of coastal wetlands within 25 years after the RSLR threshold is exceeded underscores the urgency of conservation and restoration efforts.

There is also potential to directly include TEK identified factors and data collected near dredged channels, such as digitized spoil banks and levees, into the SK model to factor in the impact of channels on vegetation and sediment dynamics. In addition, during the most recent field visit in September 2023, one of the sample sites showed a potential transition from *Spartina alterniflora* dominated coastal wetland habitat to a mix of *S. alterniflora*, *Salicornia bigelovii* Torr. (dwarf glasswort), and *Avicennia germinans* (black mangrove). The current model does not account for vegetation community change so it is recommended to alter the model to factor it in this. Further visits to this site will potentially show continued presence of these new species or others, which can provide more insight for the model’s landscape predictions.

Likewise, the presence of the glasswort and mangrove species may be indicative of ecological succession – as climate change continues, these new species may continue to expand poleward into territory previously dominated by *Spartina alterniflora*. This expansion of black mangroves into the Terrebonne Bay could affect landscapes of this region dramatically, further challenge spatial predictions, and potentially reshape restoration priorities as a forward-looking adaptation challenge. Mangroves may be more tolerant to increased inundation than *S. alterniflora* as they can build elevation more rapidly, and are already being used to increase shoreline protection along coastal Louisiana (Morris et al., 2023; Yao et al., 2022; Madison et al., 2013). However, these plants can collapse abruptly due to quick decomposition of necromass after mortality (Morris et al., 2023). In addition, mangroves, while somewhat resilient to cold freezes, are more susceptible to freezing events than *S. alterniflora* (Yao et al., 2022; Osland et al., 2017; McKee et al., 2004; Alleman & Hester, 2011). The literature suggests more research is needed to understand what ecological consequences and emerging implications may arise from this shift in Louisiana’s coastal wetland habitats (Perry & Mendelssohn, 2009; Guo et al., 2013).

The effort to compare the PACIT’s TEK assessments and the biophysical, mechanistic predictions of the SK model highlighted the complementarity of the datasets. Overall, there are observable areas of alignment between the biophysical predictions and the TEK assessments, particularly the TEK sustainability assessment (Fig. 8a-b). Some of the discrepancies between the RSLR model and the TEK vulnerability assessment can be attributed mainly to different views of RSLR scenarios, highlighting the values of both knowledge systems, particularly over time (Tsuji & Ho, 2002; Eijck & Roth, 2007). Time played a large role in the differences between the vulnerability assessments, as the SK vulnerability describes future predicted loss by various years while the TEK vulnerability highlights vulnerable areas identified by the tribe at one point in time but informed by past events with a coarse estimate of future RSLR.

Additionally, the TEK assessment considered risk factors such as proximity to spoil banks, canals, and highly fragmented areas, which were not considered in the SK model. These cultural and anthropogenic factors in the TEK assessment, and absent from SK, underscores added value. In contrast, while the original TEK vulnerability assessment takes into account historical land loss, it does not account for future land loss at a site-specific accelerating RSLR rate, as seen in the RSLR SK model. Lastly, the TEK assessments incorporated site-specific areas of historical loss and sustainability factors that can guide preservation priorities, while the RSLR model’s inputs represent a larger spatial area (biophysical data). With these differences in mind, it is important to note that the TEK assessments are fundamentally highlighting the PACIT’s knowledge based on past experiences and current conditions on where to take action, whereas the RSLR predictions provide a timeframe for when to take future action. The comparison shows the two systems are not redundant but complementary with both discrepancy and consistency in this specific scenario, offering a different but useful combined decision-support opportunity as the comparisons allow a dual-lense approach for wetland management and restoration in this region.

Working within the PACIT community provided a alternative perspective on the RSLR issue, detailing a completely new piece to the climate change puzzle that SK traditionally ignores and highlighting the importance of including traditional and social data (Gadgil et al., 1993; Berkes, 1993; Molnár & Babai, 2021). As impacts from climate change worsen, the knowledge systems from TEK provide generational familiarity of previous ecological changes, which can lend insight for successful adaptation plans (Maldonado, 2014). One of the initial goals of this collaboration initiated by Dr. Bethel was not only to aid the PACIT’s planning for hazard mitigation, but to serve as a means of communicating their perspectives and needs to external partners and government agencies for consideration in protective and restorative projects (Bethel et al., 2022). These results indicate a pathway for making combined community-driven data systems the norm, not the exception in coastal restoration and adaptation planning processes.

In creating this decision support tool through previously collected TEK spatial data and SK vulnerability assessments, the Tribe’s members have a more informed means for assessing climate change impacts of the territory they’ve lived and worked in for generations, allowing better preparation and adaptation to a changing landscape while protecting their sites of cultural significance (Maldonado, 2014; Bethel et al., 2022). By comparing and aligning a biophysical SK model with the TEK assessments, local management may be able to better tackle the resiliency concerns of these coastal wetlands and preserve the PACIT’s way of life (Molnár & Babai, 2021; Choudhury et al., 2021). The results based on high acceleration scenario (worse scenario) indicate that much of the larger wetland loss won’t occur until mid-century, suggesting that these coastal wetland systems can be resilient to some sea level rise, despite an accelerating RSLR, suggesting there is time to implement wetland restoration and mitigate wetland loss. The varied wetland loss scenarios suggest that restoration efforts, depending on accretion in particular, are viable methods to mitigate wetland degradation. Restoration efforts should be guided by the previously co-produced TEK assessments and complemented by the SK model. This means locating targetable priority areas that are addressable through informed, intensive restorative projects, while bearing in mind that restoration success is largely dependent on enhancing accretion rates. This combined assessment may help the PACIT and land managers prioritize limited resources to target areas that have the greatest potential to be protected over a simulated timeframe. By incorporating generational and cultural data into the landscape predictions, broader perspectives were considered, which are often missing from the limited biophysical, mechanistic models (Hatfield et al., 2018).

## Conclusion

Spatial analysis comparing TEK and SK model predictions allowed a comprehensive understanding of the PACIT’s wetland vulnerability from historical, current, and future perspectives. The TEK and SK approaches offer complementary assessments—TEK highlights social-cultural vulnerabilities and sustainability priorities provided by the PACIT, while the SK model produced predictive temporal scenarios. The comparisons between the SK model and TEK assessments showed where areas aligned and disagreed, emphasizing sustainability factors contributing to lower vulnerability and identifying missing factors in the separate vulnerability assessments. The TEK vulnerability assessment consisted mostly of medium to low/no priority areas, influenced by the PACIT’s perception of risk, while the predictive SK vulnerability map demonstrated a large section of high to medium priority, likely influenced by its biophysical inputs and scenario implemented.

Working with the PACIT and their TEK provided critical information that bolstered the SK interpretations to be more relatable and relevant for climate adaptation; this combined tool will continue to lend insight to local decision-making as climate change exacerbates changes in this landscape. As the SK model predicts major RSLR-driven wetland loss between 2050 and 2075, there is a clear window of time for restoration based on the TEK priorities, keeping in mind that restoration success is directly impacted by increasing accretion rates. The co-produced GIS tool supports PACIT decision-making and could be considered a potential model for Indigenous-led resilience planning, with the caveat that TEK data is inherently place-specific and culturally specific, with assessment replication largely dependent on the TEK inputs that may be different for other communities. Therefore the comparative approach demonstrated here should be adapted with Tribes in other contexts rather than directly replicated. Through this tool, the PACIT and local land managers can better understand how this region of the Terrebonne Bay is changing and identify what areas should be protected first based on prioritization of goals. Additionally, continued field site visits are encouraged to observe potential ecological succession and to examine areas close to dredged channels to potentially include anthropogenic influence, increasing awareness for the landscape predictions, and to strengthen the bond forged with the PACIT community.

## Supplementary Information

Below is the link to the electronic supplementary material.

ESM 1 DOCX (1.64 MB)

## Data Availability

The datasets during and/or analyzed during the current study are available from the corresponding author on reasonable request.
