# Supplementary material for: Assessing Resilience of a Coastal Wetland to Relative Sea-level Rise for a Native American Tribe in Louisiana – Comparing Biophysical Prediction and Traditional Ecological Knowledge
Source: Estuaries Coast. 2026 Feb 20;49(3):57. doi: 10.1007/s12237-026-01679-5 (PMC12923504; doi:10.1007/s12237-026-01679-5)
Supplement: Supplementary file 1 — DOCX (1.64 MB) [file 12237_2026_1679_MOESM1_ESM.docx]

ASSESSING RESILIENCE OF A COASTAL WETLAND TO RELATIVE SEA-LEVEL RISE FOR A NATIVE AMERICAN TRIBE IN LOUISIANA – COMPARING BIOPHYSICAL PREDICTION AND TRADITIONAL ECOLOGICAL KNOWLEDGE

Kelly M. San Antonio 1*†, Wei Wu 1, Matthew B. Bethel 2

1School of Ocean Science & Engineering, The University of Southern Mississippi, 703 East Beach Dr., Ocean Springs, MS 39564, USA

2Louisiana Sea Grant College Program, Louisiana State University, 232 Sea Grant Building, Baton Rouge, LA, 70803, USA

*Author to whom correspondence should be addressed.

†Current address: Department of Integrated Environmental Science, Bethune-Cookman University, Daytona Beach, FL 32114, USA.

**Electronic Supplemental Material**


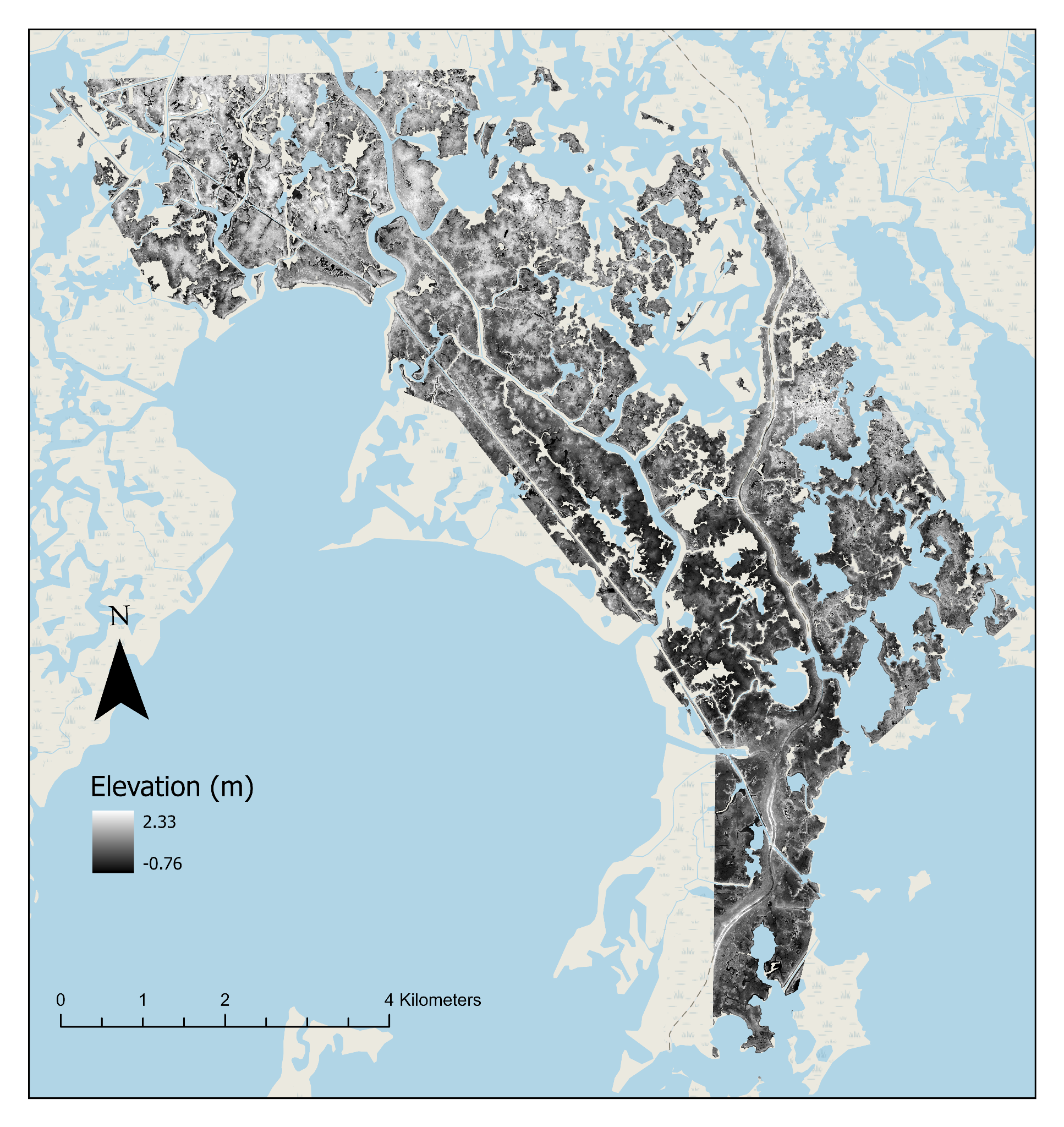


**Fig. S1** Elevation of the study area in meters using 2011 LiDAR data.

**Table S1** SLR scenario simulations by hectare area.

| Year | Accelerating SLR 9 - 14 mm | Doubled Accretion, Halved Erosion | Halved Erosion | Doubled Accretion | Accretion * 1.2 | Accretion * 1.4 | Accretion * 1.6 | Accretion * 1.8 | Accretion * 1.9 |
| --- | --- | --- | --- | --- | --- | --- | --- | --- | --- |
| 2018 | 2537.31 | 2539.22 | 2539.22 | 2537.31 | 2537.31 | 2537.31 | 2537.31 | 2537.31 | 2537.31 |
| 2019 | 2529.96 | 2537.31 | 2536.05 | 2532.47 | 2530.11 | 2531.86 | 2532.47 | 2532.47 | 2532.47 |
| 2020 | 2517.73 | 2534.52 | 2532.47 | 2525.90 | 2520.71 | 2522.56 | 2522.74 | 2523.12 | 2524.41 |
| 2021 | 2502.70 | 2532.35 | 2527.02 | 2516.64 | 2503.39 | 2509.71 | 2511.04 | 2512.21 | 2515.33 |
| 2022 | 2480.27 | 2529.50 | 2522.24 | 2503.64 | 2482.62 | 2491.99 | 2494.26 | 2501.71 | 2502.68 |
| 2023 | 2451.15 | 2526.33 | 2512.13 | 2492.94 | 2465.25 | 2469.97 | 2480.36 | 2486.51 | 2491.52 |
| 2024 | 2435.42 | 2522.78 | 2503.52 | 2481.21 | 2442.54 | 2451.92 | 2465.22 | 2472.58 | 2479.34 |
| 2025 | 2408.27 | 2520.61 | 2492.97 | 2468.83 | 2421.94 | 2438.04 | 2449.73 | 2459.41 | 2466.64 |
| 2026 | 2384.38 | 2517.14 | 2481.17 | 2460.25 | 2402.11 | 2419.78 | 2437.38 | 2449.99 | 2452.67 |
| 2027 | 2365.43 | 2514.66 | 2467.79 | 2451.11 | 2385.80 | 2404.81 | 2423.03 | 2438.86 | 2446.05 |
| 2028 | 2343.64 | 2511.23 | 2453.89 | 2444.28 | 2369.46 | 2393.61 | 2414.87 | 2432.38 | 2437.88 |
| 2029 | 2323.82 | 2509.93 | 2444.37 | 2438.47 | 2351.01 | 2376.01 | 2399.37 | 2420.54 | 2432.80 |
| 2030 | 2311.71 | 2506.74 | 2436.48 | 2435.06 | 2340.87 | 2368.66 | 2393.42 | 2415.62 | 2423.55 |
| 2031 | 2295.15 | 2504.75 | 2420.09 | 2427.22 | 2324.61 | 2354.21 | 2381.65 | 2405.98 | 2417.82 |
| 2032 | 2285.85 | 2502.52 | 2409.46 | 2422.53 | 2317.26 | 2346.13 | 2373.66 | 2399.11 | 2413.69 |
| 2033 | 2271.97 | 2501.37 | 2395.94 | 2418.74 | 2305.21 | 2339.33 | 2368.80 | 2395.04 | 2406.78 |
| 2034 | 2262.47 | 2499.79 | 2383.27 | 2415.41 | 2295.31 | 2328.89 | 2361.76 | 2390.89 | 2402.61 |
| 2035 | 2256.53 | 2497.50 | 2372.15 | 2411.77 | 2289.50 | 2321.79 | 2355.31 | 2385.30 | 2398.64 |
| 2036 | 2245.98 | 2496.12 | 2360.18 | 2408.47 | 2283.97 | 2318.15 | 2349.53 | 2381.38 | 2395.55 |
| 2037 | 2237.61 | 2495.48 | 2348.28 | 2405.37 | 2275.12 | 2313.37 | 2346.36 | 2377.54 | 2392.85 |
| 2038 | 2231.46 | 2494.47 | 2337.99 | 2403.78 | 2268.32 | 2307.19 | 2342.75 | 2374.80 | 2389.86 |
| 2039 | 2225.82 | 2493.14 | 2325.83 | 2402.06 | 2264.22 | 2301.98 | 2339.06 | 2372.36 | 2387.19 |
| 2040 | 2215.41 | 2492.19 | 2315.84 | 2400.28 | 2260.73 | 2297.45 | 2335.50 | 2369.83 | 2384.66 |
| 2041 | 2204.84 | 2491.54 | 2305.20 | 2398.51 | 2258.02 | 2293.93 | 2332.39 | 2367.79 | 2382.74 |
| 2042 | 2190.94 | 2490.91 | 2295.22 | 2397.22 | 2253.83 | 2291.89 | 2329.96 | 2365.43 | 2381.65 |
| 2043 | 2176.09 | 2490.15 | 2285.04 | 2396.11 | 2248.87 | 2289.87 | 2327.42 | 2363.48 | 2380.58 |
| 2044 | 2155.55 | 2489.06 | 2273.85 | 2394.99 | 2243.96 | 2286.93 | 2325.16 | 2361.84 | 2379.62 |
| 2045 | 2131.48 | 2488.24 | 2265.14 | 2394.08 | 2238.91 | 2284.91 | 2323.43 | 2360.13 | 2378.44 |
| 2046 | 2099.64 | 2487.54 | 2254.48 | 2393.15 | 2233.47 | 2282.82 | 2322.25 | 2358.83 | 2377.47 |
| 2047 | 2058.13 | 2486.87 | 2242.27 | 2392.28 | 2226.50 | 2280.43 | 2320.95 | 2357.77 | 2376.36 |
| 2048 | 2007.28 | 2486.30 | 2231.26 | 2391.47 | 2217.01 | 2278.23 | 2319.44 | 2357.21 | 2375.32 |
| 2049 | 1948.13 | 2485.67 | 2217.28 | 2390.56 | 2205.08 | 2276.15 | 2318.05 | 2356.64 | 2374.46 |
| 2050 | 1880.67 | 2485.21 | 2198.94 | 2389.82 | 2186.13 | 2274.37 | 2316.89 | 2356.00 | 2373.74 |
| 2051 | 1808.41 | 2484.92 | 2176.84 | 2389.19 | 2159.58 | 2272.60 | 2315.91 | 2355.35 | 2373.14 |
| 2052 | 1734.35 | 2484.71 | 2149.95 | 2388.65 | 2122.34 | 2270.44 | 2315.07 | 2354.91 | 2372.63 |
| 2053 | 1657.87 | 2484.49 | 2113.85 | 2388.18 | 2071.33 | 2267.18 | 2314.09 | 2354.47 | 2372.25 |
| 2054 | 1581.30 | 2484.34 | 2067.77 | 2387.77 | 2008.15 | 2262.45 | 2313.16 | 2353.92 | 2371.88 |
| 2055 | 1506.83 | 2484.23 | 2010.71 | 2387.42 | 1933.76 | 2255.29 | 2312.32 | 2353.44 | 2371.58 |
| 2056 | 1431.49 | 2484.13 | 1944.52 | 2387.05 | 1851.26 | 2244.24 | 2311.55 | 2352.97 | 2371.32 |
| 2057 | 1359.53 | 2484.06 | 1871.64 | 2386.75 | 1764.05 | 2227.37 | 2310.78 | 2352.61 | 2371.08 |
| 2058 | 1285.70 | 2483.97 | 1793.65 | 2386.47 | 1675.39 | 2200.89 | 2310.00 | 2352.24 | 2370.83 |
| 2059 | 1216.26 | 2483.90 | 1713.51 | 2386.22 | 1587.24 | 2163.62 | 2309.09 | 2351.85 | 2370.63 |
| 2060 | 1147.28 | 2483.83 | 1632.86 | 2386.01 | 1499.77 | 2113.31 | 2307.88 | 2351.51 | 2370.38 |
| 2061 | 1080.26 | 2483.74 | 1551.55 | 2385.83 | 1412.23 | 2051.75 | 2305.98 | 2351.23 | 2370.13 |
| 2062 | 1016.73 | 2483.66 | 1471.07 | 2385.66 | 1325.11 | 1978.30 | 2302.73 | 2350.97 | 2369.89 |
| 2063 | 954.97 | 2483.57 | 1392.11 | 2385.52 | 1239.31 | 1892.27 | 2297.16 | 2350.73 | 2369.69 |
| 2064 | 895.82 | 2483.49 | 1315.68 | 2385.39 | 1157.64 | 1799.48 | 2288.10 | 2350.48 | 2369.50 |
| 2065 | 840.83 | 2483.43 | 1240.87 | 2385.28 | 1080.60 | 1705.34 | 2274.49 | 2350.25 | 2369.36 |
| 2066 | 784.37 | 2483.35 | 1168.92 | 2385.18 | 1008.36 | 1611.09 | 2254.41 | 2350.05 | 2369.21 |
| 2067 | 733.75 | 2483.28 | 1099.45 | 2385.08 | 939.93 | 1517.13 | 2224.72 | 2349.87 | 2369.09 |
| 2068 | 680.97 | 2483.21 | 1032.12 | 2385.03 | 876.73 | 1426.17 | 2186.77 | 2349.71 | 2368.97 |
| 2069 | 633.30 | 2483.14 | 967.14 | 2384.95 | 814.85 | 1342.68 | 2143.67 | 2349.50 | 2368.85 |
| 2070 | 585.66 | 2483.07 | 905.33 | 2384.89 | 757.18 | 1263.95 | 2094.11 | 2349.27 | 2368.76 |
| 2071 | 539.19 | 2483.01 | 846.74 | 2384.84 | 702.42 | 1187.99 | 2039.32 | 2348.77 | 2368.67 |
| 2072 | 496.00 | 2482.95 | 789.07 | 2384.78 | 649.71 | 1113.70 | 1983.42 | 2347.43 | 2368.61 |
| 2073 | 450.47 | 2482.90 | 735.20 | 2384.74 | 600.76 | 1042.83 | 1928.43 | 2344.90 | 2368.53 |
| 2074 | 410.47 | 2482.85 | 682.22 | 2384.68 | 551.17 | 976.46 | 1872.72 | 2341.13 | 2368.48 |
| 2075 | 369.16 | 2482.79 | 631.79 | 2384.65 | 504.99 | 910.47 | 1810.77 | 2335.51 | 2368.44 |
| 2076 | 330.31 | 2482.76 | 584.31 | 2384.61 | 460.63 | 847.32 | 1743.30 | 2327.87 | 2368.39 |
| 2077 | 294.87 | 2482.71 | 536.58 | 2384.58 | 417.36 | 785.35 | 1671.73 | 2318.45 | 2368.35 |
| 2078 | 257.57 | 2482.67 | 492.01 | 2384.55 | 376.98 | 725.02 | 1600.56 | 2307.64 | 2368.31 |
| 2079 | 226.18 | 2482.63 | 447.50 | 2384.52 | 335.86 | 667.34 | 1530.26 | 2295.21 | 2368.26 |
| 2080 | 194.33 | 2482.60 | 405.26 | 2384.50 | 298.56 | 610.43 | 1460.81 | 2281.50 | 2368.07 |
| 2081 | 166.22 | 2482.56 | 365.17 | 2384.47 | 262.54 | 556.48 | 1392.52 | 2266.70 | 2367.56 |
| 2082 | 140.94 | 2482.52 | 325.37 | 2384.45 | 228.18 | 502.38 | 1325.21 | 2249.88 | 2366.36 |
| 2083 | 116.77 | 2482.50 | 289.49 | 2384.42 | 197.69 | 450.93 | 1262.52 | 2230.60 | 2364.29 |
| 2084 | 97.83 | 2482.47 | 252.93 | 2384.42 | 167.27 | 402.36 | 1201.84 | 2207.75 | 2361.68 |
| 2085 | 79.54 | 2482.45 | 220.02 | 2384.39 | 142.16 | 355.28 | 1143.72 | 2181.84 | 2358.64 |
| 2086 | 64.72 | 2482.43 | 189.65 | 2384.38 | 118.34 | 312.19 | 1087.82 | 2154.31 | 2355.04 |
| 2087 | 51.83 | 2482.40 | 160.41 | 2384.36 | 97.78 | 270.60 | 1034.95 | 2124.21 | 2350.37 |
| 2088 | 41.18 | 2482.38 | 136.60 | 2384.36 | 81.00 | 232.24 | 984.11 | 2092.29 | 2344.66 |
| 2089 | 33.18 | 2482.36 | 112.42 | 2384.33 | 64.42 | 198.45 | 934.20 | 2059.27 | 2337.85 |
| 2090 | 25.95 | 2482.34 | 93.72 | 2384.33 | 52.67 | 167.65 | 886.06 | 2025.86 | 2330.00 |
| 2091 | 20.60 | 2482.32 | 76.72 | 2384.33 | 41.94 | 141.37 | 839.80 | 1992.24 | 2321.31 |
| 2092 | 16.21 | 2482.31 | 61.13 | 2384.33 | 33.33 | 118.18 | 794.60 | 1959.01 | 2311.75 |
| 2093 | 12.85 | 2482.28 | 50.23 | 2384.31 | 26.93 | 98.46 | 750.21 | 1926.54 | 2301.71 |
| 2094 | 10.47 | 2482.26 | 39.09 | 2384.31 | 20.89 | 82.22 | 708.77 | 1894.81 | 2291.01 |
| 2095 | 8.46 | 2482.24 | 31.63 | 2384.31 | 17.03 | 67.87 | 669.29 | 1863.35 | 2279.59 |
| 2096 | 7.03 | 2482.23 | 25.19 | 2384.31 | 13.61 | 56.02 | 633.02 | 1831.69 | 2267.91 |
| 2097 | 5.94 | 2482.22 | 19.59 | 2384.30 | 10.98 | 45.68 | 599.03 | 1800.05 | 2255.64 |
| 2098 | 5.02 | 2482.20 | 16.15 | 2384.28 | 9.16 | 37.29 | 568.16 | 1767.82 | 2243.13 |
| 2099 | 4.44 | 2482.19 | 12.54 | 2384.28 | 7.60 | 30.74 | 540.97 | 1735.43 | 2230.43 |
| 2100 | 3.93 | 2482.18 | 10.27 | 2384.28 | 6.52 | 25.31 | 516.01 | 1703.76 | 2217.41 |
